# Therapeutic Approaches for Toxic Optic Neuropathies: Insights from Methanol-Induced Optic Neuropathy and NAION Treatments

**DOI:** 10.3390/diagnostics15222883

**Published:** 2025-11-13

**Authors:** Lorenzo Verriello, Giada Pauletto, Marco Zeppieri, Simone Lorenzut, Chiara Bertolotti, Caterina Gagliano, Fabiana D’Esposito, Matteo Capobianco, Marieme Khouyyi

**Affiliations:** 1 Neurology Unit, Head Neck and Neurosciences Department, Santa Maria della Misericordia University Hospital, Piazzale Santa Maria della Misericordia 15, 33100 Udine, Italy; lorenzo.verriello@asufc.sanita.fvg.it (L.V.); giada.pauletto@asufc.sanita.fvg.it (G.P.);; 2 Department of Ophthalmology, University Hospital of Udine, 33100 Udine, Italy; 3 Department of Medicine, Surgery and Health Sciences, University of Trieste, 34127 Trieste, Italy; 4 Department of Neurology, University of Trento, 38121 Trento, Italy; 5 Department of Medicine and Surgery, University of Enna “Kore”, Piazza dell’Università, 94100 Enna, Italy; 6 Eye Center “G.B. Morgagni-DSV”, 95125 Catania, Italy; 7 Imperial College Ophthalmic Research Group [ICORG] Unit, Imperial College, London NW1 5QH, UK; 8 Eye Clinic, Catania University, Policlinico G. Rodolico, Via Santa Sofia, 95121 Catania, Italy; 9 Faculty of Medicine, University of Catania, 95123 Catania, Italy; 10 Department of Biomedical and Dental Sciences and Morphofunctional Imaging, University of Messina, 98121 Messina, Italy

**Keywords:** progressive vision loss, dyschromatopsia, optic nerve pallor, mitochondrial dysfunction, oxidative stress, neuroprotective agents

## Abstract

**Background/Objectives**: Toxic optic neuropathy (TON) represents a spectrum of optic nerve damage caused by exposure to toxins, including drugs, alcohol, and industrial chemicals. It is characterized by progressive vision loss, dyschromatopsia, and optic nerve pallor and poses a clinical challenge in diagnosis and management due to overlapping features with other optic neuropathies. Non-arteritic anterior ischemic optic neuropathy (NAION), although distinct, shares common pathophysiological mechanisms such as oxidative stress and mitochondrial dysfunction. This review aims to evaluate therapeutic strategies applied in TON and discuss the potential role of NAION-targeted treatments in TON management. **Methods**: We reviewed medical therapies previously used in NAION patients, including corticosteroids and neuroprotective substances, and analyzed their relevance in the context of TON. Particular focus was given to emerging interventions targeting oxidative stress and mitochondrial health, including experimental drugs. **Results**: Evidence indicates that early diagnosis and toxin removal are essential in preventing irreversible vision impairment in TON. Therapies for methanol-induced and drug-related ocular neuropathies have demonstrated inconsistent efficacy, especially when integrated with antioxidant and neuroprotective approaches. However, the search for potential synergy between detoxification protocols and NAION-targeted treatments offers a promising direction for comprehensive management strategies. **Conclusions**: While current therapeutic options remain controversial and often unsatisfactory, integrating detoxification with interventions aimed at oxidative stress and mitochondrial function may improve outcomes. Further research is needed to develop targeted therapies for TON and bridge gaps in clinical decision-making.

## 1. Introduction

Optic neuropathies represent a heterogeneous group of disorders characterized by dysfunction of the optic nerve, leading to vision loss. These conditions can arise from ischemic, inflammatory, demyelinating, infectious, nutritional, or toxic causes, posing a diagnostic and therapeutic challenge in neuro-ophthalmology. Toxic optic neuropathy (TON) comprises a group of clinical entities characterized by progressive bilateral and symmetrical vision loss, often associated with central or centrocecal scotomas. TONs may result from exposure to toxic substances in occupational settings, ingestion of contaminated materials, or systemic medication use. Among the drugs most commonly associated with TON development, there are amiodarone, anti-tubercular agents, linezolid, and phosphodiesterase type 5 inhibitors [1,2].

Amiodarone, a widely prescribed antiarrhythmic agent, causes optic neuropathy in approximately 2% of users, with visual loss typically manifesting within one year of treatment initiation. Anti-tubercular drugs, particularly ethambutol, induce optic neuropathy in a dose-dependent manner by accumulating in retinal ganglion cells (RGCs), which leads to a calcium imbalance and the generation of reactive oxygen species (ROS). Isoniazid can also cause optic neuropathy, though less frequently and with greater reversibility. Linezolid, an antibiotic for resistant Gram-positive infections, may lead to optic neuropathy after prolonged therapy [1,2]. Phosphodiesterase type 5 inhibitors, such as sildenafil, vardenafil, and tadalafil, have been linked to probable instances of non-arteritic anterior ischemic optic neuropathy (NAION). Evidence indicates that these drugs may trigger a vascular event in susceptible individuals rather than causing a true toxic optic neuropathy. Consequently, its association with TON is indirect [1,2].

Among TON caused by toxin ingestion, methanol-related optic neuropathy is the most common. Its metabolites, such as formaldehyde and formic acid, induce ganglion cell and optic nerve necrosis through oxidative stress and mitochondrial dysfunction [3]. Methanol is found in industrial solvents, cleaners, and antifreeze; exposure may occur accidentally or, rarely, intentionally [4]. Heavy metal exposure—particularly to cobalt, arsenic, lead, or cadmium—can also lead to optic neuropathy and neurological damage [5]. Tobacco–alcohol amblyopia is another form of TON associated with both nutritional deficiencies and the direct toxic effects of nicotine and alcohol [5].

The underlying pathogenic mechanisms of TON are primarily linked to mitochondrial damage, which triggers oxidative stress and the subsequent death of RGCs [6]. Experimental studies indicate that secondary inflammatory responses may exacerbate neuronal damage, after mitochondrial dysfunction, potentially leading to progressive degeneration alongside ischemic phenomena [7].

Non-arteritic anterior ischemic optic neuropathy (NAION) is primarily ascribed to hypoperfusion at a congested optic nerve head, resulting in axoplasmic flow stasis, mitochondrial energy deficiency, and the buildup of reactive oxygen species. These processes initiate further inflammatory cascades and the apoptotic demise of retinal ganglion cells. The interplay of oxidative stress, mitochondrial failure, and inflammation creates a molecular connection between NAION and toxic ocular neuropathies, where direct toxic or drug-induced mitochondrial damage similarly undermines ganglion-cell viability [6,7]. The common biological foundation warrants a comparative comparison of therapy techniques between the two entities.

This review summarizes current therapeutic approaches for TON and evaluates the potential applicability of NAION-targeted therapies to improve management and patient outcomes.

## 2. Materials and Methods

We conducted an extensive literature review using PubMed, Scopus, and Web of Science to identify pertinent English-language publications on optic neuropathies secondary to toxic factors. The principal search strings comprised combinations of the following terms: (‘toxic optic neuropathy’ OR ‘methanol optic neuropathy’ OR ‘drug-induced optic neuropathy’) AND (‘treatment’ OR ‘therapy’ OR ‘corticosteroids’ OR ‘erythropoietin’ OR ‘antioxidants’ OR ‘neuroprotection’). Boolean operators were customized for each database. The search primarily included studies published between 2000 and 2025; however, earlier seminal papers were also considered when historically or mechanistically relevant Only peer-reviewed original research articles, reviews, and clinical studies in English were considered. Duplicates and non-relevant papers were excluded after title and abstract screening. This review follows a narrative, non-systematic approach, aiming to summarize current evidence and highlight areas for future research.

All data discussed in this article were obtained from publicly available sources. No human or animal experiments were conducted, and no ethical approval was required.

## 3. Toxic Optic Neuropathy Pathophysiology

TON is caused by damage to the axons of ganglion cells in the papillomacular bundle, with pathophysiological mechanisms varying depending on the specific toxic substance involved. However, the mitochondria of retinal ganglion cells (RGCs), particularly in the papillomacular bundle, appear to be common targets for many causes of TON. RGCs have one of the highest energy demands in the body and a high oxygen consumption rate, rendering them particularly vulnerable to mitochondrial dysfunction. Toxic agents or their metabolites interfere with oxidative phosphorylation in the mitochondria, disrupting energy production necessary for normal cellular function. This interference leads to an accumulation of reactive oxygen species (ROS), which can damage lipids, proteins, and DNA, contributing to oxidative stress.

The accumulation of ROS and the resulting energy depletion cause significant oxidative stress in the cells, which can further damage cellular structure and function. Oxidative stress and energy depletion activate apoptosis, characterized by the release of cytochrome C from the mitochondria, initiating signaling cascades that lead to the death of RGCs [3]. The papillomacular bundle is especially sensitive due to its densely packed, small-caliber retinal ganglion cell axons with high energy demand, which makes them more physiologically vulnerable to mitochondrial impairment. The absence of myelin results in a higher energetic request; thus, reduced energy production increases susceptibility to damage. In summary, the pathogenic mechanisms underlying TON revolve around mitochondrial damage, leading to a cycle of oxidative stress and cellular injury, ultimately culminating in the death of RGCs and the loss of visual function [1,3].

## 4. Clinical Presentation

Patients with TON typically present with bilateral, painless, progressive vision loss, often accompanied by central or centrocecal scotomas, while peripheral vision is usually preserved. A change in color perception (dyschromatopsia) is frequently one of the initial symptoms. Patients may observe that certain colors, particularly red, appear less vivid [2].

## 5. Diagnostic Methods

The diagnosis of TON relies on a combination of a detailed medical history and a thorough ocular examination [2]. A comprehensive patient history is crucial for identifying potential exposures to toxic substances, whether through environmental factors, diet, or medication use. This may include detailed inquiries about the patient’s occupation, dietary habits, and alcohol consumption. A complete eye examination, including visual field testing, can help identify any central or centrocecal scotomas. The optic disc may appear normal, swollen, or hyperemic in the early stages, with optic atrophy and temporal pallor developing in chronic stages. Optical coherence tomography (OCT) serves as an effective tool for quantifying early changes related to toxicity, even before fundus alterations become visible [2,8,9].

Initial thinning typically occurs in the inferotemporal sector of the papillomacular bundle [10,11,12], followed by a reduction in the thickness of the retinal nerve fiber layer across all quadrants in the later stages [13]. In specific cases, laboratory tests may be required to detect the presence of toxins in blood or urine, such as methanol levels. If heavy metal exposure is suspected, specific tests for lead, thallium, and other metals are conducted. Although imaging studies typically yield normal results, magnetic resonance imaging of the optic nerve and chiasm, with and without gadolinium, may be indicated to rule out other causes. These combined elements help confirm the diagnosis of TON and guide treatment and patient management.

## 6. Therapeutic Approaches

The therapeutic options for TON vary depending on the underlying causative factor. The following paragraphs synthetize the main treatments, according to optic neuropathy etiology.

### 6.1. Antidotal Therapy and Detoxification

For TON due to medications or exposure to toxic substances, the primary treatment consists of discontinuing exposure to the suspected drug or toxin. In many cases, this can lead to an improvement in visual function, although complete reversibility is not always achieved. In methanol-induced optic neuropathy (MION), caused by the accumulation of formic acid—a toxic metabolite of methanol—leading to mitochondrial damage and degeneration of RGCs, conventional treatment primarily involves the use of ethanol or fomepizole as antidotes, along with bicarbonate, folate, and, if necessary, extracorporeal elimination, predominantly through hemodialysis [4]. Ethanol and fomepizole work by preventing the conversion of methanol to formic acid, thereby halting the further production of toxins. Bicarbonate helps neutralize acidosis, while folate promotes the conversion of formic acid to carbon dioxide and water. Extracorporeal elimination facilitates the removal of methanol and its toxic metabolites from the body [4].

### 6.2. Anti-Inflammatory Agents and Corticosteroids

For MION, studies support the use of corticosteroids and recombinant human erythropoietin (EPO), administered either alone or in combination with systemic corticosteroid therapy. Glucocorticoids are well-known for their ability to reduce the production of pro-inflammatory cytokines, enhance the synthesis of anti-inflammatory molecules, and exhibit neuroprotective and anti-demyelinating properties. Various treatment regimens have been reported, including intravenous administration of 1 g of methylprednisolone per day for 3–4 days [3,14,15]. Notably, significant improvements in visual acuity have been reported following corticosteroid treatment. For example, in the study by Abrishami et al. involving six patients, high-dose steroids were evaluated for the treatment of methanol-induced optic neuropathy [14]. Patients received 250 mg of methylprednisolone intravenously every 6 h for 4 days, followed by oral prednisolone at 1 mg/kg for 10 days. Best-corrected visual acuity (BCVA), OCT, fundus photography, and a comprehensive ophthalmological examination were conducted before and three months after treatment, showing a significant improvement in BCVA [14]. The authors concluded that high-dose intravenous methylprednisolone may provide benefits in MION. However, the absence of a control group limits the accuracy of assessing corticosteroid efficacy. Subsequent to intravenous administration of methylprednisolone (1 g/day for 3–4 days), therapy is generally maintained with oral prednisolone at a dosage of 1 mg/kg/day for a duration of up to 10 days. This protocol reflects conventional neuro-ophthalmic practice. Corticosteroid therapy should be limited to certain cases in critically ill patients due to the risks of immunosuppression and sepsis, following thorough clinical evaluation [16]. Consequently, the role of corticosteroids in MION remains debated due to the lack of robust clinical evidence [17,18].

Considering NAION, it does not have a broadly recognized disease-modifying treatment. The present care emphasizes immediate distinction from other ocular neuropathies, guidance on natural history, and the enhancement of modifiable vascular risk factors, including nocturnal hypotension, diabetes, dyslipidemia, and tobacco use. Corticosteroid therapy is contentious; whereas multiple non-randomized studies indicate temporary enhancement in visual function or expedited clearance of optic-disc edema, randomized controlled trials have not substantiated a consistent advantage [16]. Antiplatelet and anticoagulant treatments have been utilized predominantly for systemic vascular protection rather than demonstrated ocular effectiveness. Experimental methodologies encompass the utilization of neuroprotective and antioxidant agents designed to stabilize mitochondrial energy metabolism, diminish oxidative stress, and avert apoptosis. Erythropoietin, citicoline, nicotinamide, and CoQ10 analogues have demonstrated neuroprotective effects in preclinical or limited investigations; nevertheless, substantial clinical evidence remains insufficient. Consequently, the management of NAION primarily relies on the control of risk factors and personalized supportive therapy.

### 6.3. Erythropoietin and Neuroprotective Agents

EPO is a glycoprotein known for increasing red blood cell mass by preventing apoptosis of hematopoietic precursors. Beyond its hematopoietic role, numerous experimental studies have indicated that EPO may possess anti-inflammatory [19], anti-apoptotic [20], and neuroprotective properties [21,22]. In particular, EPO may provide cellular protection through intracellular antioxidant mechanisms, such as the recruitment of glutathione and heme oxygenase-1, and may indirectly stimulate iron depletion, inhibiting iron-dependent oxidative damage. Research on EPO in various ophthalmological conditions, such as ischemic retinal diseases, protection of RGCs [23], and optic neuropathies [22,23], has revealed promising therapeutic potential [24]. Overall, these findings suggest that combining detoxification, neuroprotective, and anti-inflammatory strategies could improve outcomes in patients with toxic optic neuropathies.

In a prospective study involving 16 patients diagnosed with MION, treatment with 20,000 units of EPO administered intravenously for three consecutive days resulted in a significant improvement in visual acuity [25]. In another case series, three male patients with bilateral blindness secondary to MION received low doses of subcutaneous EPO after 3 to 4 days of ineffective standard treatments. Complete recovery of visual acuity was observed within 10 to 12 days of initiating therapy [26]. In a prospective study of 105 patients who experienced bilateral visual impairment due to methanol toxicity, EPO was administered intravenously at a dosage of 10,000 IU/mL every 12 h for three consecutive days. The results indicated a significant improvement in visual acuity, with no adverse effects reported [27].

The study conducted by Alrobaian et al. assessed the effectiveness of erythropoietin (EPO) in treating toxic optic neuropathy caused by methanol by using a retrospective analysis [28]. It included nine patients, five of whom were treated with EPO in addition to conventional management, while four received only conventional therapy. Patients receiving EPO were treated with 20,000 IU for three consecutive days. Despite this treatment, no significant difference in visual outcomes was observed between the EPO and non-EPO groups. Final visual measurements showed similar values in both groups. OCT analysis indicated thinning of the retinal nerve fiber layer in patients treated with EPO, suggesting progressive damage to the optic nerve. Although one patient exhibited a significant temporary improvement in vision, this was followed by deterioration, highlighting the potentially transient nature of EPO effects [28].

Some studies support the use of EPO in combination with systemic corticosteroid therapy in MION. Zamani et al. performed a case–control study to assess the effectiveness of EPO in association with corticosteroids compared to corticosteroids alone for the treatment of MION [29]. The study included a total of 15 patients, comprising 10 cases and 5 controls. All control patients reported improvements in visual acuity after discharge, while only three patients in the case group noted similar enhancements. Additionally, some individuals in the EPO treatment group experienced initial improvements in visual acuity, which were later followed by a decline in their visual acuity. At follow-up, the control group exhibited superior visual acuity compared to the case group. The authors concluded that EPO may provide an initial protective effect, but this effect appears to be transient in cases of MION [29].

In the study by Pakravan et al., the impact of EPO combined with methylprednisolone was assessed, and the results were compared to those of a historical control group that received only standard treatment [30]. This non-randomized study involved 22 participants, 11 of whom received EPO treatment (10,000 IU twice a day for three days) along with methylprednisolone, while the other 11 constituted the historical control group. Participants were followed for a variable period, with the monitoring of EPO side effects, including increased blood pressure and elevated red blood cell counts. Results demonstrated a significant improvement in BCVA in the EPO group compared to the control group. Additionally, retinal nerve fiber layer thickness decreased in both groups; however, it remained significantly thicker in the EPO group at the final follow-up. No significant side effects associated with EPO use were reported [30]. The study involved a limited number of participants, which restricts the generalizability of the findings. Furthermore, the absence of randomization may have introduced bias into the results. Finally, using a historical control group instead of a contemporaneous parallel control group may affect the comparability of the outcomes.

In the study carried out by Badeeb et al., the effectiveness of EPO in treating MION was assessed using a series of retrospective case analyses [31]. The research focused on patients diagnosed with this condition between November 2022 and December 2023 at two centers in Jeddah, Saudi Arabia. Within this case series, EPO, in association with steroids, demonstrated variable effects on visual improvement. Although some visual enhancements were observed post-treatment, these differences did not reach statistical significance [31].

A recent systematic review investigated the potential therapeutic role of EPO in treating MION [32]. Among the 139 studies identified, 11 were included in the final analysis, encompassing a total of 212 participants, with 192 receiving EPO treatment. Improvements in visual acuity were observed in 184 patients, with outcomes ranging from complete recovery of vision to minor enhancements. Only 8 patients did not exhibit any changes or experienced a decline in their condition. Additionally, a reduction in the thickness of the retinal nerve fiber layer was observed in 21 cases, with one case returning to the normal range. The review highlights the positive effects of EPO on visual acuity in patients with MION [32]. Nonetheless, the simultaneous administration of EPO and corticosteroids in studies without control groups complicates the evaluation of erythropoietin’s independent effectiveness. The discrepancies in reported outcomes among EPO trials seem to arise from diversity in treatment schedule, dose, and patient selection. Timely administration within 72 h post-toxin exposure and elevated cumulative dosages (≥20,000 IU over three days) have typically resulted in more favorable outcomes than delayed or subcutaneous treatments. Moreover, variations in initial severity, the absence of randomization, and the lack of uniform visual endpoints lead to inconsistent outcomes. Future controlled trials utilizing standardized EPO methods and stratified patient groups are necessary to elucidate its actual usefulness in TON [18]. Finally, erythropoietin has been studied in ischemic ocular neuropathies, such as NAION, due to its potential neuroprotective and anti-apoptotic effects that may mitigate ganglion-cell loss, in addition to its role in toxic optic neuropathies. Minor interventional and observational studies have indicated temporary enhancements in visual acuity and field sensitivity; however, variability in dosage, delivery method, and timing, together with the lack of extensive controlled trials, has hindered conclusive findings. Consequently, whereas EPO signifies a potential pathway for neuroprotection in toxic and ischemic ocular neuropathies, its therapeutic effectiveness in NAION has yet to be determined.

### 6.4. Mitochondrial Support Therapies and Antioxidants

Additionally, studies have been conducted using antioxidant molecules to improve the prognosis of MION. Antioxidants could theoretically be utilized to reduce oxidative stress caused by the accumulation of ROS in the mitochondria. This approach may help mitigate cellular damage. Among the antioxidant molecules, TEMPOL (4-hydroxy-TEMPO), a low-molecular-weight cyclic nitroxide with excellent cellular permeability, serves as a superoxide dismutase (SOD) mimic. In addition to its antioxidant activity, TEMPOL exhibits anti-apoptotic, anti-inflammatory, and immunomodulatory properties [33,34,35,36,37,38,39].

In the study by Setiohadji et al., the efficacy of TEMPOL in improving the cellular structure of RGCs in methanol-intoxicated rats was evaluated [40]. The research was conducted on 20 male Wistar rats divided into four groups: negative control, positive control, methanol group, and methanol + TEMPOL. Enucleated eyes were analyzed histologically to assess cellular structure, swelling, and vacuole formation in the ganglion cells of the retina. TEMPOL demonstrated a significant positive effect on the structure of RGCs in methanol-intoxicated rats, suggesting that antioxidant therapy could represent a promising therapeutic option for MION [40]. The study focused on a relatively short observation period, which limited its understanding of TEMPOL’s long-term effects. Additionally, the reduced number of animals per group may limit the reliability of the results. While the findings are promising in animal models, caution is warranted when translating them directly to human clinical practice due to interspecies differences in methanol metabolism.

Another molecule with antioxidant properties is rutin, a bioflavonoid found in various plants, including those from the Rutaceae family. This substance is attributed several beneficial properties, justifying its inclusion in different dietary supplements. Its most significant capability is to promote normal permeability of blood capillaries, counteracting the formation of edema. Rutin also appears to stimulate trophism and the elasticity of blood capillaries, enhancing circulation and preventing hemorrhages. Generally, rutin-based products are employed in the treatment of hemorrhoids and the alleviation of symptoms associated with circulatory disorders in the lower limbs (edema, varicose veins, itching).

Several studies have also highlighted the antioxidant properties of this substance, which can protect cells from the harmful effects of free radicals; moreover, rutin can prevent blood clot formation due to its antiplatelet and antithrombotic properties. In the study by Taşlı et al., the protective effects of rutin in acute MION were evaluated, comparing the results with those of ethanol [41]. The research involved 30 male Wistar albino rats, divided into five groups: healthy control (C), methotrexate (MTX), methotrexate + methanol (MTM), methotrexate + methanol + ethanol (MTME), and methotrexate + methanol + rutin (MTMR). Rats in the MTM, MTME, and MTMR groups received methanol following treatment with methotrexate for 7 days. Ethanol was administered in the MTME group, while rutin was given in the MTMR group. In this study, the authors also investigated tissue levels of 8-hydroxy-2-deoxyguanosine (8-OHdG) as a determinant of DNA damage; inflammatory markers such as interleukin-1β (IL-1β) and tumor necrosis factor-alpha (TNF-α); oxidative stress parameters including myeloperoxidase (MPO) and malondialdehyde (MDA); as well as antioxidants like glutathione peroxidase (tGSH) and superoxide dismutase (SOD) to clarify the mechanism of association. Significant changes were observed in the levels of 8-OHdG, IL-1β, TNF-α, MDA, MPO, tGSH, and SOD among the groups. The MTMR group exhibited levels comparable to those of the healthy controls, suggesting that rutin can prevent the increase in oxidative stress and inflammation. Additionally, histopathological analysis indicated that the optic nerve tissue in the MTMR group resembled that of healthy controls, demonstrating a protective effect of rutin. Notably, the MTM group exhibited the most severe pathological alterations, while the MTME group showed moderate changes [41]. The study suggests that rutin is effective in reducing markers of oxidative stress and inflammation, improving antioxidant levels in rats with methanol toxicity, and may hold potential as a treatment to prevent MION. However, the follow-up period was short, limiting the understanding of the long-term effects of rutin. Furthermore, the limited number of rats in each group may not represent a wide range of biological responses, and results obtained from animal models may not be fully applicable to humans.

Another molecule that may play a role in managing TONs is coenzyme Q10, also known as ubiquinone, a lipophilic molecule present in cell membranes and mitochondria that supports mitochondrial energy production. Its ability to reduce oxidative stress and improve mitochondrial function may protect RGCs from the toxic effects of drugs or toxins. In a study conducted by Irma et al., the protective role of coenzyme Q10 (CoQ10) against ethambutol-induced toxicity in RGCs in mice was evaluated [42]. The study was designed as a randomized, triple-blind trial involving 18 mice divided into a treatment group and a control group. All mice received 25 mg/kg of ethambutol daily, while the treatment group additionally received 100 mg/kg of CoQ10. After 30 days, histological analyses revealed that the mice treated with CoQ10 exhibited a significantly higher density of RGCs compared to the control mice. Statistically, this translates to an average of 47.2 cells per microscopic field in the treatment group compared to 33.5 in the control group, with a *p*-value of 0.004, indicating a statistically significant difference [42]. These results suggest that CoQ10 may protect RGCs from ethambutol-induced toxicity, which could have implications for the treatment of human patients suffering from tuberculosis and treated with ethambutol. However, the study had several limitations, including a small sample size and the absence of an established optimal dosage for CoQ10. Additionally, while the results in mice are promising, caution is warranted when translating findings from animal models to humans due to biological differences. Finally, the relatively short duration of the study (30 days) may not be sufficient to observe the long-term effects of CoQ10 treatment.

In a study by Nalcacioglu et al., the therapeutic effects of methylprednisolone and idebenone, a structural analogue of CoQ10, were evaluated in relation to methanol-induced toxic damage to the optic nerve and retina in rats [43]. The research involved 30 male Wistar rats, which were categorized into five groups based on their treatment protocols: a healthy control group (HC), a methanol group (M), a methanol + methylprednisolone group (MM), a methanol + idebenone group (MI), and a methanol + methylprednisolone + idebenone group (MMI). All rats, except those in the control group, received methanol orally. The MM group was treated with intraperitoneal methylprednisolone for 10 days, while the MI group received oral idebenone for 28 days. The MMI group received both treatments at the same dosages. At the end of the treatment, serum samples were collected for biochemical analysis, and the rats were sacrificed for histopathological examination of their eyes. The findings showed no statistically significant differences in oxidative stress biomarkers among the groups. However, histopathological analysis indicated that the MMI group showed the most important improvements in optic nerve circumference thickness, vascularization, and number of astrocytes compared to the other groups [43].

In summary, the early administration of idebenone, combined with short-term methylprednisolone treatment, appears to provide protective effects against optic nerve and retinal damage following methanol ingestion in rats, as supported by histopathological findings.

In the context of NAION, antioxidant and mitochondrial-support drugs have been regarded as potential neuroprotective adjuncts [44]. Preclinical NAION models have shown decreased retinal ganglion-cell apoptosis and enhanced mitochondrial bioenergetics after the administration of superoxide dismutase mimetics like TEMPOL, bioflavonoids such as rutin, and mitochondrial cofactors such coenzyme Q10 and its counterpart idebenone. While these findings offer a mechanical justification for translation, clinical data in human NAION are still nascent and predominantly anecdotal. No randomized controlled research has conclusively shown a benefit; hence, these interventions should now be regarded as experimental alternatives within the larger context of oxidative-stress management.

### 6.5. Ethambutol-Induced Optic Neuropathy (EB-TON)

Management focuses on the prompt withdrawal of the drug upon suspicion of toxicity, as ongoing exposure has been linked to the exacerbation of retinal ganglion cell damage. Reduction in dosage alone has been deemed inadequate in patients exhibiting evident structural or functional impairments. Visual recovery is more probable with brief exposure and timely termination; nonetheless, partial or no recovery has been observed following extended treatment durations or diminished renal clearance. Supportive therapy has involved the optimization of the anti-tubercular regimen in collaboration with infectious disease specialists, rectification of nutritional deficiencies, and meticulous structural and functional monitoring utilizing optical coherence tomography and automated perimetry at four- to eight-week intervals during the initial three to six months post-cessation.

The efficacy of corticosteroids in EB-TON remains ambiguous, and their routine application is not substantiated by controlled evidence. Considering the mitochondrial pathophysiology and oxidative stress associated with drug-induced optic neuropathies, supplementary mitochondrial support with coenzyme Q10 or idebenone, along with antioxidant supplementation, has been evaluated individually, recognizing the scarcity of robust human evidence. Experimental and small animal investigations indicate that coenzyme Q10 exposure may preserve retinal ganglion cells in cases of ethambutol toxicity, hence providing a foundation for translational research in meticulously regulated clinical environments. In conclusion, the prompt cessation of ethambutol has been fundamental to treatment, although supplementary mitochondrial and antioxidant approaches have emerged as potential, yet still experimental, additions [45].

### 6.6. Linezolid-Induced Optic Neuropathy (LZ-TON)

Linezolid has impeded mitochondrial protein synthesis, and chronic exposure has been associated with an increased risk of ocular neuropathy. The principal therapeutic intervention involves the immediate cessation of linezolid or the transition to an alternate antibiotic upon suspicion of toxicity, preferably in collaboration with the treating infectious disease team and taking into account therapeutic drug monitoring where feasible. Enhancements in color vision and central scotomas frequently manifest within weeks to several months post-discontinuation; however, factors such as delayed onset, extended exposure, renal dysfunction, and concurrent nutritional deficiencies have been linked to incomplete recovery.

Corticosteroids have not been assigned a definitive role in LZ-TON. Due to the focus on mitochondrial dysfunction, mitochondrial support and antioxidant treatments have been deemed appropriate adjuncts for certain patients, accompanied by thorough counseling of the inadequate evidence strength. A routine follow-up using visual fields and macular and peripapillary OCT has been advised to record early stabilization and to measure subsequent recovery [46].

### 6.7. Amiodarone-Induced Optic Neuropathy (AMIO-ON)

Amiodarone-induced optic neuropathy has shown as a gradual deterioration of vision, often accompanied by optic disc edema that can endure for several months. Proposed causes include drug-induced mitochondrial dysfunction and possible microvascular ischemic effects, which have coincided with the phenomenology of non-arteritic anterior ischemic optic neuropathy in certain patients. Drug withdrawal has been the primary option when practicable, following collaborative decision-making with cardiology, including the risk of arrhythmia recurrence. In instances where withdrawal was unfeasible, dosage reduction and enhanced ocular monitoring have been implemented.

The evidence endorsing corticosteroid therapy has been inadequate, and routine steroid administration is not advised. Adjunctive neuroprotection and antioxidant strategies have been evaluated on an individualized basis due to the mitochondrial role in pathogenesis while recognizing the lack of randomized trials [1,2]. The prognosis has been inconsistent, with improved outcomes noted when amiodarone is discontinued early and in the absence of pre-existing ‘disc-at-risk’ architecture or significant vascular comorbidities.

### 6.8. Nutritional and Tobacco-Alcohol Optic Neuropathy

Therapeutic interventions involve the cessation of alcohol and tobacco consumption, organized nutritional rehabilitation, and the supplementation of B-complex vitamins, particularly thiamine, cobalamin, and folate [5]. Visual enhancement typically necessitates many weeks to months and is contingent upon the duration of exposure and the severity of initial deficiencies. Due to the association of mitochondrial dysfunction and oxidative stress, supplementary antioxidant support has been contemplated, however, conclusive evidence remains scarce. Timely recognition and comprehensive interdisciplinary assistance for addiction and nutrition have proved important to enduring recovery.

### 6.9. Optic Neuropathy Associated with Heavy Metal Exposure

Upon suspicion of exposure to lead, thallium, cobalt, arsenic, or cadmium, management has involved source elimination, occupational health assessment, and toxicology-directed chelation in conjunction with a clinical toxicologist [1,2]. The selection of chelating agent and time has been contingent upon the particular metal burden and systemic condition. Supportive ophthalmic therapy has encompassed dietary enhancement, antioxidant interventions, and sequential OCT and visual field assessments to measure stabilization. The visual prognosis has fluctuated according on the severity and duration of exposure, as well as the promptness of management.

An overview of etiologies, diagnostic features, and therapeutic strategies is summarized in Table 1 and Table 2.

## 7. Link to NAION

Collectively, these etiology-specific strategies have underscored that although conceptual parallels with NAION have warranted investigation into anti-inflammatory and mitochondrial-targeted neuroprotection, the pivotal therapeutic intervention across non-methanol toxic optic neuropathies has consistently been the prompt identification and elimination of the causative agent, accompanied by adjunctive measures customized to the predominant mechanism and the patient’s comorbidity profile.

## 8. Future Innovation

Future research should aim to standardize objective endpoints and risk stratification using OCT-angiography of the macula and optic disc with serial follow-up, as already explored in prospective MION cohorts and proposed as quantitative biomarkers of disease progression and therapeutic response [47]. Moreover, the conduct and completion of randomized controlled trials on EPO with homogeneous protocols (dose, timing, and duration) are warranted, as indicated by ongoing registries and preliminary RCT experiences in MION [48,49]. Finally, building on the broader literature on optic nerve mitochondrial neuroprotection (e.g., nicotinamide, citicoline, CoQ derivatives/idebenone, mitochondrial-targeted peptides), translational and proof-of-concept studies could clarify whether such strategies improve visual outcomes in MION in addition to causal therapy and antidotes [50].

## 9. Conclusions

This review provides a detailed overview of the pathogenic mechanisms, clinical manifestations, and therapeutic options related to TONs. These conditions, characterized by progressive bilateral and symmetrical vision loss, result from damage to the axons of RGCs, particularly in the papillomacular bundle. Mitochondrial dysfunction has emerged as a common pathogenic denominator, with many toxic substances interfering with oxidative phosphorylation in the mitochondria, leading to oxidative stress and cellular damage that culminate in the death of RGCs and the loss of visual function. Clinically, the diagnosis of TONs relies on an accurate medical history and a comprehensive ophthalmological examination, often supported by laboratory tests and imaging to rule out other causes. Early identification of exposure to toxic substances is crucial for improving prognosis, as the cessation of contact with the harmful substance can lead to an improvement in visual function. However, complete reversibility of the damage cannot always be guaranteed.

In terms of treatment, therapeutic strategies vary depending on the underlying cause. For TONs induced by drugs or other substances, discontinuing the causal agent is the first step to preventing further damage. The treatment of MION has shown promising results with the use of antidotes, such as ethanol and fomepizole, which prevent the conversion of methanol into formic acid, a toxic metabolite. Additionally, considering the pathophysiological similarities with NAION, the use of EPO and antioxidant therapies may be considered as potential additional treatments, offering protection against oxidative and apoptotic damage. However, available studies are often limited by a small sample size and lack of randomization, highlighting the need for further research to confirm the efficacy of these therapies and optimize treatment protocols.

In conclusion, the therapeutic landscape of TONs is complex and requires a personalized approach based on the specific etiology and severity of optic nerve damage. While significant progress has been made in understanding the pathogenic mechanisms and potential therapies, it remains essential to continue conducting rigorous clinical studies to improve the management and treatment of these debilitating conditions.

## Figures and Tables

**Table 1:** Etiologies of toxic optic neuropathy and key diagnostic features.

Etiology/Toxin	Mechanism of Toxicity	Typical Clinical Features	Diagnostic Findings
**Amiodarone**	Mitochondrial dysfunction and possible microvascular ischemic mechanism	Progressive, often insidious vision loss within 1 year of therapy	Optic disc swelling, VF defects, history of amiodarone
**PDE5 inhibitors (sildenafil, vardenafil, tadalafil)**	Suspected vascular dysregulation; possible NAION-like mechanism	Sudden vision loss, usually unilateral	VF defects resembling NAION; causal link debated
**Anti-tubercular drugs (ethambutol, isoniazid)**	Ethambutol: dose-dependent accumulation in RGCs→Ca^2+^ imbalance, ROS generation; Isoniazid: reversible mitochondrial effect	Bilateral, painless, progressive loss; central/cecocentral scotomas, dyschromatopsia	OCT: early inferotemporal PMB thinning→later diffuse RNFL loss; VF central defects
**Linezolid**	Mitochondrial protein synthesis inhibition	Vision loss after prolonged use (months)	Optic disc pallor, VF central scotoma; history of long-term linezolid
**Methanol**	Metabolism→formic acid→mitochondrial dysfunction, ROS, necrosis of RGCs	Bilateral, acute–subacute severe loss, central scotomas, dyschromatopsia	Optic disc hyperemia→atrophy; OCT: PMB thinning; confirm methanol in blood
**Heavy metals (lead, thallium, cobalt, arsenic, cadmium)**	Direct neurotoxicity, mitochondrial impairment	Progressive visual loss ± systemic neuro signs	History of exposure; lab tests for metals; OCT RNFL thinning
**Tobacco–alcohol amblyopia**	Combined nutritional deficiency + toxic effects of nicotine/ethanol	Bilateral, gradual, painless vision loss, dyschromatopsia (red–green), central scotomas	Temporal disc pallor; VF central/cecocentral loss; history of alcohol/tobacco abuse

Abbreviations: RGC = retinal ganglion cell; PMB = papillomacular bundle; RNFL = retinal nerve fiber layer; OCT = optical coherence tomography; VF = visual field.

**Table 2:** Therapeutic strategies in toxic optic neuropathy and evidence summary.

Therapy	Mechanism/Rationale	Study Evidence	Key Findings/Limitations
**Cessation of alcohol/tobacco + vitamin supplementation**	Removes toxin exposure; addresses nutritional deficits	Clinical practice [5]	Some recovery is possible; prognosis depends on exposure duration
**Methanol antidotes (ethanol, fomepizole, bicarbonate, folate, hemodialysis)**	Block conversion of methanol → formic acid; correct acidosis; enhance toxin elimination	Classic protocols [4]	Effective if early; limited once optic nerve damage is established
**Corticosteroids (IV methylprednisolone)**	Anti-inflammatory, neuroprotective	Case series (Abrishami 2011, Sharma 2012, Kowalski 2019) [14,15,16,17]	Mixed outcomes; possible benefit if early; risk of sepsis/immunosuppression; lack of randomized controlled trials
**Erythropoietin (EPO)**	Anti-apoptotic, antioxidant, neuroprotective	Prospective/retrospective studies [25,26,27,28,29,30,31,32]	Some improvement in BCVA; results inconsistent; effect may be transient; RCTs lacking
**EPO + corticosteroids**	Synergistic neuroprotection	Zamani 2018, Pakravan 2016, Badeeb 2024 [29,30,31]	Some benefit in BCVA and RNFL thickness; limited by small samples, non-randomized designs; Conflicting results across studies; variability in dosage and timing
**TEMPOL (superoxide dismutase mimetic)**	Antioxidant, anti-apoptotic, anti-inflammatory	Animal model (Setiohadji 2018) [40]	Improved RGC morphology; short follow-up; translation to humans uncertain
**Rutin (bioflavonoid)**	Antioxidant, anti-inflammatory, vascular protective	Rat study (Taşlı 2018) [41]	Reduced oxidative stress markers; protective histology; short-term, small sample
**Coenzyme Q10**	Mitochondrial support, antioxidant	Mouse RCT (Irma 2022) [42]	Preserved RGC density in ethambutol toxicity; small sample, limited duration
**Idebenone + corticosteroids**	CoQ10 analogue + anti-inflammatory synergy	Rat model (Nalcacioglu 2022) [43]	Histological protection; no biomarker differences; animal-only evidence

Abbreviations: EPO = erythropoietin; BCVA = best-corrected visual acuity; RNFL = retinal nerve fiber layer; RGC = retinal ganglion cell.

## Data Availability

No new data were created or analyzed in this study. Data sharing is not applicable to this article.
